# Abortion policy implementation in Ireland: Lessons from the community model of care

**DOI:** 10.1371/journal.pone.0264494

**Published:** 2022-05-09

**Authors:** Joanna Mishtal, Karli Reeves, Dyuti Chakravarty, Lorraine Grimes, Bianca Stifani, Wendy Chavkin, Deirdre Duffy, Mary Favier, Patricia Horgan, Mark Murphy, Antonella F. Lavelanet

**Affiliations:** 1 Department of Anthropology, University of Central Florida, Orlando, Florida, United States of America; 2 Department of Population Health Sciences, University of Central Florida, Orlando, Florida, United States of America; 3 School of Sociology, University College Dublin, Dublin, Republic of Ireland; 4 Department of History, National University of Ireland Galway, and Social Sciences Institute, Maynooth University, Kildare, Republic of Ireland; 5 Department of Obstetrics and Gynecology, New York Medical College/Westchester Medical Center, New York, New York, United States of America; 6 Global Doctors for Choice, New York, New York, United States of America; 7 Department of Social Work and Social Care, Manchester Metropolitan University, Manchester, United Kingdom; 8 Parklands Surgery, Cork, Republic of Ireland; 9 Broad Lane Family Practice, Cork, Republic of Ireland; 10 Eldon Family Practice, Dublin, Republic of Ireland; 11 UNDP-UNFPA-UNICEF-WHO-World Bank Special Programme of Research, Development and Research Training in Human Reproduction (HRP), Department of Sexual and Reproductive Health and Research, World Health Organization, Geneva, Switzerland; Northumbria University, UNITED KINGDOM

## Abstract

**Background:**

In 2018, the right to lawful abortion in the Republic of Ireland significantly expanded, and service provision commenced on 1 January, 2019. Community provision of early medical abortion to 9 weeks plus 6 days gestation delivered by General Practitioners constitutes the backbone of the Irish abortion policy implementation. We conducted a study in 2020–2021 to examine the barriers and facilitators of the Irish abortion policy implementation.

**Methods:**

We collected data using qualitative in-depth interviews (IDIs) which were conducted in-person or remotely. We coded and analysed interview transcripts following the grounded theory approach.

**Results:**

We collected 108 IDIs in Ireland from May 2020 to March 2021. This article draws on 79 IDIs with three participant samples directly relevant to the community model of care: (a) 27 key informants involved in the abortion policy development and implementation representing government healthcare administration, medical professionals, and advocacy organisations, (b) 22 healthcare providers involved in abortion provision in community settings, and (c) 30 service users who sought abortion services in 2020. Facilitators of community-based abortion provision have been: a collaborative approach between the Irish government and the medical community to develop the model of care, and strong support systems for providers. The MyOptions helpline for service users is a successful national referral model. The main barriers to provision are the mandatory 3-day wait, unclear or slow referral pathways from primary to hospital care, barriers for migrants, and a shortage and incomplete geographic distribution of providers, especially in rural areas.

**Conclusions:**

We conclude that access to abortion care in Ireland has been greatly expanded since the policy implementation in 2019. The community delivery of care and the national helpline constitute key features of the Irish abortion policy implementation that could be duplicated in other contexts and countries. Several challenges to full abortion policy implementation remain.

## Introduction

Prior to 2018, abortion was banned in almost all circumstances in Ireland. Abortion was regulated under the 1861 Offences Against the Person Act, and the 1983 Eighth Amendment of the Irish Constitution further restricted abortion access by giving equal status to the life of the woman and to that of the fetus [1, 2]. The 2013 Protection of Life During Pregnancy Act slightly liberalised access to care in emergency circumstances. Following a popular referendum on 25 May, 2018, abortion became lawful in Ireland under certain circumstances. After years of advocacy work led by key women’s, civil society, and doctors’ advocacy organizations [3], the Eighth Amendment was repealed by a margin of 66.4% to 33.6% [4]. On 20 December, 2018, the Health (Regulation of Termination of Pregnancy) Act took effect, which allows abortion on request for pregnancies less than 12 weeks of gestation. It also allows abortion services until fetal viability for maternal health risks to health or life, including in emergency, as well as in cases of fatal fetal abnormality [5]. Abortion remains criminalised if performed outside of the legal parameters with sanctions of up 14 years imprisonment hanging over doctors, but criminalisation does not apply to women obtaining abortions.

Services commenced on 1 January, 2019. The model of care for women seeking an abortion under 12 weeks gestation (known as early medical abortion, EMA) consists of 3 consultations, with a mandatory 3-day wait between the first and second visits. Abortion is provided by General Practitioners (GPs) in their regular practices across Ireland, and five family planning clinics in County Dublin. By clinical guidance, GPs provide only medical abortion, and up to 9 weeks plus 6 days gestation. Cases from 10 weeks to 12 weeks plus 0 days are managed in secondary care in hospitals within maternity units by Obstetricians and Gynaecologists (OB/GYNs), where medical or surgical abortion can be offered, depending on local availability of services. Midwives and nurses have a significant role in facilitating abortion care. Access to care is also facilitated by a national 24/7 helpline provided by the Irish public healthcare system, the Health Service Executive (HSE), called MyOptions where women and other pregnant people may obtain information about providers, counselling, and clinical nursing supports [6]. There were 6666 abortions provided in 2019 in Ireland, and 6577 in 2020; 98% (6542 and 6455 respectively) were in the first trimester [7, 8]. The vast majority of abortion provision takes place in primary care in the community [9].

Scholarship on abortion provision shows that primary care may be a useful site to maximise availability of abortion, especially access to early medical abortion [10]. The implementation of the Australian community model with abortion services available in primary care delivered by GPs and nurses, has been hailed by abortion care experts as a “one stop shop in their own community” [11], which has greatly increased access in rural areas and significantly decreased time and travel for patients [12]. A study conducted in the United States (US) showed that women generally preferred to receive early abortion from their primary provider with whom they have an established relationship, improving continuity of care for these patients [13].

The Irish community model for EMA constitutes an important example of an implementation of this service via primary care. The purpose of this paper is to report findings from a research study conducted in 2020–2021 related to the implementation of the abortion policy in Ireland, with the focus on the implementation of abortion provision in the community by GPs. Our approach was to gain a holistic understanding of the community provision by bringing together interview data from three perspectives: key informants at the HSE and advocacy organisations who have been involved in the development, and implementation of the model of care leading to the start of services on 1 January, 2019; GPs who have conceptualised the model, and undertaken the implementation and service provision; and women who have sought abortion services in Ireland. While our overall policy implementation study utilised mixed-methods, in this paper we focus on the qualitative data. We use qualitative research and grounded theory inductive analysis [14] to understand the motivations and strategies used in the design of the community model of care, and the subsequent experiences on the ground from providers and users. This analysis has the potential to inform community provision of abortion care in other sociocultural and geopolitical contexts.

## Research methods

### Recruitment and ethics

In the overall study, we collected 108 qualitative IDIs in Ireland from May 2020 to March 2021, however this article draws on 79 interviews that are directly relevant to the community model of care. (We do not present interview data with hospital providers and patients, as these data are outside of this article’s scope.) The 79 IDIs were collected with three samples of participants using purposive sampling [15]. The first sample consisted of key informants: public health administrators, medical professionals who contributed to the implementation process, and reproductive health advocacy organisations. We recruited key informants via direct contact with relevant HSE offices and stakeholder organisations, including some with whom we had previous contact. The second sample consisted of primary healthcare providers in communities. We recruited community providers through an announcement mailout to the Irish College of General Practitioners [16] membership, and through a WhatsApp (a text and video messaging platform) provider network.

Our third sample consisted of service users: those women who were 18-years-old or above and sought abortion services in Ireland in 2020 were eligible to participate. We recruited service users through open advertisement on three social media platforms (Twitter, Facebook, and Instagram). Twenty-three GP offices across Ireland also distributed study flyers.

We acknowledge that transgender and gender nonbinary people may also get pregnant and seek abortion care, therefore, for the remainder of the article, when using the term women we have an inclusive intent to denote women and pregnant people.

This study received ethical approval by the World Health Organisation Research Ethics Review Committee (protocol ID: A66001; approved 17 March 2020), and the University of Central Florida (UCF) Institutional Review Board (protocol ID: 00000846; approved 11 October 2019). The study did not deviate from the approved protocol. At the time of research design and data collection for this study, there was no federal review of social research required in Ireland, and Irish research regulation did not require an ethical approval from an Irish institution for this work [17, 18], therefore the WHO and UCF approvals were sufficient to meet local requirements. A scheme for a future national-level review system in Ireland was proposed in 2019, but this bill remains inactive as primary legislation is yet to be drafted [18].

### Research team, data collection, and analysis

Our study team comprised 11 researchers in the fields of social sciences, public health and policy, and medicine. Four team members experienced in systematic qualitative research conducted data collection and analysis related to the three samples used in this article. The principal investigator, JM (PhD anthropology), and lead research assistant, KR (MA anthropology student) were US-based. JM was based in Ireland for four months of data collection. Two research assistants based in Ireland conducted all service user IDIs: LG (PhD history, Galway), and DC (PhD sociology student, Dublin). We developed three interview guides with questions exploring participants’ experiences regarding barriers and facilitators in the abortion policy implementation from the perspectives of service provision (providers), abortion care seeking (services users), and the implementation process from administrative and advocacy viewpoints (key informants). Our guides were informed by the Contextual Interaction Theory’s three variables that have been identified as important in successful policy implementation: motivation, information, and power [19]. We conducted team training on approaches to counter potential biases during interviewing and analysis.

We conducted IDIs remotely or in-person, using interview guides. Each interview lasted 60 to 90 minutes. We observed COVID-19 guidelines for in-person interviews, which took place in government buildings, hospitals, clinics, participants’ homes, outdoor public spaces, and offices of advocacy organisations. We consented participants prior to starting interviews: key informants and providers gave written informed consent (documented via a paper or an electronic consent form). Service users gave a verbal consent for the purposes of anonymous data collection with this sample (documented electronically by the researcher). We de-identified participants, except for those who chose to be identified (Simon Harris, and Peter Boylan). Organisations which are publicly known to be participating in abortion policy implementation or advocacy have not been de-identified in the quotes. In three IDIs two key informants requested to be interviewed jointly to represent a particular HSE office, therefore some quotes are referenced with two participants. Participants did not receive remuneration.

Four team members (JM, KR, DC, LG) coded and analysed interview transcripts using Dedoose software. We double-coded approximately 10% of the transcripts in each sample to develop codebooks and ensure coding consistency. We compared codes and resolved any discrepancies, with no significant disagreements in data interpretation [20]. We then finalised the codebooks for each sample separately. We then compared the codes between samples, and extracted themes and subthemes relevant to community care implementation from all samples. The multi-step coding process included open and axial coding and followed the “dynamic and fluid process” of the grounded theory approach [14]. This approach is iterative and interpretive, and identifies both predetermined, *a priori codes* (e.g., “information flow”), as well as the emergence of inductive, not previously considered factors or explanations (e.g., “pathway to hospital care”) [21]. Completion of the coding process generated a detailed thematic dataset for each sample. The samples sizes were sufficient to permit saturation of themes [22]. The study’s results and ensuing recommendations underwent both expert checking and member checking via three dissemination meetings with the HSE and the Irish medical community. We also conducted expert checking via consultations with our Irish team members who served as local advisers in Dublin and Cork, and an external adviser to the study in Cork, Ireland. We report our results in accordance with the Standards for Reporting Qualitative Research (SRQR) recommendations (S1 Appendix) [23].

Contextual interaction theory, (CIT) which recognises that policy implementation is an interactive process of actors within a specific context, informed the interview guide development and our data analysis [19]. According to the theory, the abortion policy implementation process involves activities and interactions between several individuals and/or organisations who already work together in a variety of contexts, including government, healthcare providers, and service users. In the first section of the results, we focus on the interaction between the government and the medical community during the development of the model of care and the rollout of services. In the second section, we analyse three significant strategies that have facilitated service implementation, including during the COVID-19 pandemic. In the third section, we consider the four significant remaining challenges. We conclude with a discussion of the implications of the barriers and facilitators to the abortion policy implementation in Ireland.

## Results

### Participant characteristics

Our sample consisted of 79 participants. The key informant sample consisted of 27 participants: 13 HSE officials including the former Minister for Health Simon Harris, 10 representatives of reproductive health and justice organisations including those working with migrants and ethnic minorities, and four representatives of medical organisations involved in provision of reproductive care and advocacy. The second sample consisted of 22 primary healthcare providers in communities: 20 GPs who provide abortion care, one midwife, and one administrative coordinator for abortion services. Nineteen of these 22 providers worked in general practice, and 3 worked in sexual and reproductive health clinics. Nineteen of 20 GPs were principal doctors in their practices, and one was an assistant GP. Five GPs had been involved in the abortion policy development. There was a wide geographic distribution of providers in this study with participants located in 12 of 26 counties. All providers started participating in the service in 2019, with most beginning in January of that year. Our third sample were 30 service users (see Table 1), who came from 12 of the 26 counties, distributed across urban and rural areas. Due to the complexities of ethnic identities, we asked participants to self-describe this characteristic. All participants in this sample either received abortion care in the community or received the initial consultation with community GPs before being referred to a hospital. Twenty-two participants used the MyOptions helpline at some point in seeking abortion, 12 of whom used MyOptions as their portal of entry. Eight did not use MyOptions, but five of them were already under medical care due for anomalous fetuses and directly referred. No participants withdrew from the study.

**Table 1:** Service users’ sample characteristics.

#	County	Age	Employment	Income per week	Self-described ethnicity	Did the Service Users use MyOptions?
1	Dublin	34	Finance	“High”	White Irish	No
2	Waterford	30	Student	€200	White Irish	No
3	Galway	40	Unemployed	None	White Irish	Yes—referred by GP
4	Dublin	42	Office worker	€1200	White Irish	Yes—portal of entry
5	Dublin	47	Information technology	€1300	White Irish	No
6	Cork	40	Librarian	€600	White Irish	No
7	Mayo	30	Agriculture	€190	White European	Yes—referred by a hospital
8	Kildare	44	Counsellor	€400	White Irish	Yes—portal of entry
9	Dublin	23	Unemployed	None	Malay	Yes—referred by GP
10	Kildare	43	Unemployed	None	Irish Catholic	Yes—found MyOptions on hospital website
11	Wicklow	34	Social worker	€205	Catholic, White Irish	Yes—portal of entry
12	Dublin	26	Manager	€625	White Irish	No
13	Carlow	32	Unemployed	€260	Irish Catholic	Yes—portal of entry
14	Dublin	39	Researcher	€800	Irish	Yes—portal of entry
15	Dublin	37	Business owner	€900	White Caucasian	Yes—portal of entry
16	Cork	35	Engineer	€600	White Irish	Yes—portal of entry
17	Kildare	36	Finance	€550	White Irish	Yes—portal of entry
18	Dublin	34	Nursing	€850	White Irish	Yes—portal of entry
19	Meath	30	Unemployed	None	European	No
20	Kildare	33	Nursing	€700	White British	Yes—referred by a family planning clinic
21	Galway	27	Business consultant	€2200	Irish Catholic	Yes—referred by a family planning clinic
22	Kildare	35	Self-employed	€1000	White Irish	Yes—portal of entry
23	Kildare	28	Healthcare	€450	White Irish	Yes—referred by GP
24	Galway	28	Student	€250	White European	Yes—referred by GP
25	Kildare	32	Human resources	€900	White Irish	Yes—referred by a family planning clinic
26	Laois	38	Carer	€350	Caucasian, European-Irish	Yes—referred by GP
27	Meath	39	Teacher	€675	White Irish	Yes—portal of entry
28	Donegal	35	Administration	€420	White Irish	No
29	Tipperary	37	Humanitarian aid	€2000	White Irish	No
30	Dublin	24	Administration	€300	Irish Caucasian	Yes—portal of entry

The data obtained from these three categories of participants offer multiple perspectives on the issue of implementation that together build a holistic picture of the processes involved, in particular how the policy came about, and implementation experience related to day-to-day provision and the use of care by patients.

### I. Development and rollout of the community care model

#### General practitioners’ leadership

At the time of our study, 385 GPs provided abortion services in 25 of 26 counties in Ireland (KI3, KI4, KI26). Overall, the Irish community care model prioritised accessibility, and capitalised on the existing primary care infrastructure for EMA. GPs, many of whom were part of Doctors for Choice Ireland (DfC) [24] and/or the Southern Task-Force on Abortion and Reproductive Topics (START) advocacy organisations [25], were eager to take the lead in service provision, with preparations starting several months ahead of the May Repeal referendum. One of the GPs explained,

“we quickly realised that getting the vote passed was probably going to happen. …So we felt general practice would be ideal. You know the arguments. It’s community, it’s anonymous, it’s safe, it’s hopefully with someone they [patients] already know and respect and we have access to their medical history and their circumstances. So we kind of very quickly drafted I suppose a policy document to see how it could be done.”

(KI5)

The group was also clear about the need to prevent the establishment of a few specialised family planning clinics because of the concern that these would “silo” the service in urban centres of Dublin and Cork, and make it less accessible across other, more rural counties.

The GPs’ conceptualisation of the model and initiative resonated with the HSE. After the referendum, the finalisation of the legislation and the development of the model of care were taking place simultaneously. HSE’s departments managing community healthcare understood from “a lot of public consultation… over the years” (KI3) that there was a preference for access to EMA locally through GP clinics. Simon Harris, the Minister for Health from May 2016 to June 2020, emphasised in his interview for this study that “the GP network and the GP leadership was crucial,” specifically from the ICGP, START, the Well Woman Clinic [26], and the Irish Family Planning Association [27]. Preceding START, DfC Ireland has been the advocacy leader in this area since 2005 [28]. Accessibility was a shared goal, “firstly, at early pregnancy, that’s when most women would opt for it, and if it was an early pregnancy it made sense to try and provide it in the community in primary care outside of the hospital setting, it was safe to do and it was an effective way, and it was also a better way for the woman” (KI27). Making the service free of charge within the Irish Public Health Service was also about access, as Harris noted, “[w]e actually decided this [cost] is a barrier that can’t exist” (KI27).

Likewise, payments to GPs for services was an important aspect of the model. The negotiation of the contract for abortion care between the Irish Medical Organisation (IMO) representing GPs and the HSE resulted in a contract that provides €450 payment for a three-consultation process, which was deemed by GPs in our sample as “fair” and “appropriate,” as one doctor explains,

“…getting paid reasonably for providing the service was essential in its implementation. It just wouldn’t have happened otherwise, you know, if we were just asked to take on another thing and not be paid reasonably for. I don’t think we’re overpaid for it. I think it’s just an appropriate amount because sometimes the cases can be extremely complex and it might take up time and sometimes, you know, they can be very straightforward. But… without that remuneration, it definitely wouldn’t have happened.”

(GP4)

In addition to GPs’ interest in providing the service, several respondents also felt that Irish maternity hospitals feared being overwhelmed with the demand for abortions services. The HSE’s solution was to make primary care the portal of entry for all patients, regardless of their gestation, as one HSE representative in a leadership role at that time explained, “we wanted to make sure that women went to the GP first and were seen by the GP and then if they were over the gestation, that the GP would refer them on. So self-referral to maternity services for surgical abortion is not encouraged or wasn’t encouraged. See… your GP first, and your GP refers you.” (KI11)

Data from providers and key informants suggest that this approach was successful and that abortion care in Ireland is predominantly provided in community settings. From the perspective of the GPs, abortion care fits within the primary care scope by “normalizing” the service, and offering privacy:

“I think a key facilitator in the Irish system is that the service is available in family clinics…. You can go to my waiting room and in my waiting room, some of them are there for routine paediatrics, but… you could be there for anything’. …. But one of the real big facilitators is delivering this in the general practice setting in family practice and family practice is where this belongs…. We’re enabled to provide the service in the community close to where people live and work. So that is a key enabler, you know.”

(GP6)

The community approach also resonated with service users, as summarised by this 23-year-old woman, who emphasised the local nature of the service: the “GP… was very close to where I live. I could walk to the GP. … I didn’t experience delays” (SU9). Women expected services to be available and were surprised when their GPs were non-providers: “It would have been nice to be able to go to my GP” (SU15).

#### Collaborative effort

According to the HSE, their major effort began in the summer of 2018, and involved the Department of Health and an HSE team tasked with developing the details of the implementation. The HSE team felt “this is sensitive” and therefore “we need to make sure this is a success. This is completely new for all of us and… let’s kind of mind each other and work together through this” (KI3, KI4). Research participants from across the HSE consistently reported that they aimed for a collaborative approach by engaging stakeholders, through open input, and maintaining a constructive attitude: “So, I think we had a lot of stakeholder engagement, a lot of excellent communication and we set that tone about being inclusive” (KI3, KI4). Others confirmed,

[W]e engaged with the representative bodies, the ICGP as the training body and the kind of college, the talk leaders if you like, the IMO as the negotiating body and we worked with those collaboratively in a way, which would ensure all interests, all felt fairly dealt with, but that we’d find a mechanism of making it work, and I think we did that very well, ourselves and the Department collectively, and ourselves within the HSE, and then as entities.…[I]t was a very positive joint piece in the end.

(KI22)

The HSE team conducted stakeholder analyses to map “who should hear what when and we took all that information in then and made one big master plan, like a huge big communications plan…. So it was like break down your audience and the task and the action and when it should be completed by. It was a bit of a moveable feast sometimes, but yes” (KI8, KI9). According to the HSE, the consultations included groups representing women’s rights organisations, “vulnerable minority groups, like Traveller groups, women living in direct provision,” as well as on-going communications with provider groups such as GPs, OBGYNs, and midwives “to again flesh out the model of care even further” (KI3, KI4).

In October 2018, the then Minister for Health, Simon Harris, appointed Dr. Peter Boylan, an obstetrician and the former Master of the National Maternity Hospital in Dublin, as the national clinical adviser on the abortion policy implementation. This appointment was significant, as Harris explains, “I asked Peter to be the interlocutor if you like.…I knew that the only way you brought medics on board was medic to medic. So, Peter played a very key role in that,” and added that the role of the clinical lead was envisaged to be “a permanent feature of the Irish Health Service” (KI27). Boylan acted as the national lead during the crucial months of October 2018 until March 2019 during the rollout of services, but the post remained vacant for the rest of the year. The HSE hired a new national lead in 2020, Dr. Aoife Mullally, a practicing obstetrician, to lead the expansion of abortion services in hospitals and GP practices [29].

The GPs were central in their leadership to write the guidelines for the community care model, and finalised the document two weeks before the service launched [30]. According to Minister Harris, the condensed time period for implementing the new law helped to overcome resistance, despite “significant push back” whereby individuals wanted to “buy another few months”; he explained:

Well intentioned people and very serious people, …people who had campaigned, some leading medical experts, some Masters of our Hospitals, …I wouldn’t dismiss their view at all. But we held very firmly to the view that women couldn’t wait any longer, and actually in a weird sort of way I think a critical enabler of ensuring that the service was delivered, and delivered in a timely fashion, and that actually everybody pulled together and got on with it was the fact that we refused to budge on the introduction date of the 1^st^ January.

(KI27)

Overall, the emphasis on collaboration between the HSE and the medical and advocacy communities as a way to implement services has been a successful strategy.

### II. Three strategies for a successful implementation

#### Strategy one: “Cocooning” providers

In the initial two months of the rollout, the HSE allocated staff to field queries from GPs to resolve any problems with the new service as they arose. Queries were centralised to one email address and one phone number. Staff involved in the rollout (KI3, KI4) explained they “were able very quickly to see if there was any particular noise developing in the system or if there was any particular issue or blackspots.” The HSE mobilised the expertise of “most senior people” in the Department of Health to quickly respond to GP queries:

“we had to kind of I’ll use the word ‘cocoon’… Whatever the issue they had, we had to deal with it because we did not want them to stop providing because we had very small numbers at the start.”

(KI3, KI4)

When clinical issues emerged, including questions about failed terminations, or uncertainty about need for an ultrasound scan, the HSE mobilised several experienced GPs to ring the GP with answers.

To instill “confidence in the GPs”, the HSE staff kept in contact with each: “we actually phoned every GP who signed up…, even if we didn’t hear from them, just touching base…” asking “Is there anything that you need?… And that was really useful because a lot of them,‥ would say, ‘Oh look, I’m signed up. I’m not going to get started until I get my training from ICGP, but glad you rang. Could I have more of this?’” (KI3, KI4).

In January 2019, the queries that “caused a real amount of stress…were about women who were close to 12 weeks who were waiting because they knew the service was going live in the start of January or they were just receiving information about the service” (KI3, KI4). In these cases, the management had to be immediate: “And in some of the cases, you had the national director of acutes practically ringing around to the hospitals going, ‘Can you take this woman? Can you take her now?’.” Other study participants (GPs and OBGYNs) described that there was an initial flurry of later gestation cases as some women had been waiting to receive services in Ireland. As time went on, fewer service users presented who were close to the 12-week gestation limit, and in the first six months of 2020 there were only 30 queries overall posed by providers to the HSE, and none related to cases of patients nearing the 12-week limit. This makes sense in light of our data from GPs who reported the typical gestation for patients seeking care in 2020 to be 6–7 weeks. Other concerns expressed by GPs related to missing a clinical sign, in particular being “worried in case we’re going to miss some ectopic risk” (GP2); another remarked, “this could end in disaster. … there’s kind of the fear they could have an ectopic pregnancy and something goes wrong and you haven’t picked it up” (GP19). With each month of the service there were fewer queries, and as of June 2020, there have been no further clinical queries registered posed to the HSE, however queries continue amongst providers.

In addition to HSE’s query system, the GPs established their own voluntary peer support group that has been “cocooning” providers since the rollout. The START group formed in 2018 after the DfC Ireland organisation called a meeting of healthcare providers who supported the Repeal referendum (KI5). START, which includes mainly GPs, but also OBGYNs and midwives, subsequently expanded across Ireland; many of the group’s GPs we interviewed had no previous experience with advocacy or involvement with government but took up the effort around the time of the referendum. The group served as a central support network, runs an extensive WhatsApp network, and is independent of the ICGP, with a smaller “Train the Trainer” group who help train new providers. START continues to have a vital role, as one GP summarises, “if you have any problem or a query, whether you’re a newbie to providing the service or you’re an old hand, people throw out questions and within five minutes, you’ve got a selection of answers and advice that you can work from. It’s been amazing” (GP18). Strong peer support provided by START was also linked to feelings that the work was rewarding as evident in this emblematic excerpt:

“I suppose I find that it’s been the most rewarding work I’ve ever done, which seemed kind of incongruous in some ways, but it really has been so rewarding. …I only joined them… 1^st^ January, and they’d already been putting in all that work. And they were so welcoming. They just had such a nice can do attitude.”

(GP4)

START was intended as a temporary, informal network to help new GPs transition into providing the service, with the expectation that the network would eventually wind down and its work would be taken over and formalised by established organisations such as the ICGP, and the Institute of Obstetrics and Gynaecology (KI5). This transition has not occurred and START continues their work in 2021.

#### Strategy two: MyOptions helpline

Our data show that one of the more successful strategies facilitating access to care is the HSE’s implementation of the MyOptions freephone line [6], a national no-cost helpline staffed by experienced counsellors and nurses where women looking for abortion care in Ireland can call and receive the name and phone number for two to three specific providers near them. If they request it, women can also receive counselling during the same call. The helpline was designed to be a one-call seamless service. An interpreter service via a 3-way call is also available. The information and counselling service operates six days a week, however if a patient needs medical advice, nurses at MyOptions are also available 24/7. According to the HSE, 20–25% of callers had more than one contact/phone call with MyOptions (KI14). GPs may elect to receive referrals from MyOptions or provide services without being enlisted only to their own patients if they request them directly. The HSE considered the helpline idea early in May 2018 and contracted an experienced counselling service organisation in Ireland to run the helpline. After the referendum, it was clear the service required expansion to evenings and weekends, as HSE representatives explained:

“The 24-hour piece has been positive as well, like that, a woman at home in the middle of the night can ring a number if she’s having any difficulties. And that actually for GPs was another reassuring piece, that… if something happens…, that there is this person at the other end of the phone who can manage that at three in the morning…. I’m still very amazed that we pulled that one [MyOptions] off and that it has worked really, really well.”

(KI3, KI4)

The written and verbal communication materials developed for MyOptions services was created with inputs from an organisation supporting migrants and from “women’s groups that we[re]…consulted as part of the implementation” (KI11). The HSE also conducted focus groups as a way of “ensuring that the message was appropriate.” One of their findings was to shift from the previously used language of ‘crisis pregnancy’ to ‘unplanned pregnancy’ (KI8, KI9).

HSE and MyOptions produce an information booklet for patients which are distributed to all GPs who contract to provide abortion services. In May 2019, MyOptions added a webchat on their website to reach younger or hearing impaired people for general information and counselling, but callers still must telephone to get providers’ contact details.

From the perspectives of doctors, MyOptions is a well-functioning, core service. In addition to increasing patient flow and ensuring privacy, the availability of clinical support from trained nurses reassures GPs about care quality and patient safety as one GP, who works in an urban area in the east of Ireland explains:

“But then if they [patients] don’t know what to do over the weekend, I’m not readily available either, so My Options is their stopgap if they’re kind of worried about their bleeding or that they haven’t bled or they’re having pain, they’re able to ring MyOptions for advice as well.”

(GP18)

From the perspective of service users in our study, interactions with MyOptions were vital for accessing information about locations of care and non-judgemental support. MyOptions gives callers contact information for providers that suit their needs; this may be a doctor near them, or one who is further away if the caller prefers to avoid local GPs due to privacy concerns. Overall, the experience with the helpline was deemed excellent with comments such as, “MyOptions was brilliant” (SU26), “MyOptions woman was wonderful” (SU7). Other comments reflected specifically on the supportive aspect of the experience, for example: “As soon as I rang MyOptions she was so helpful. I felt so much better after speaking to her” (SU22).

While service users reported overwhelmingly positive experiences when interacting with MyOptions, 18 of 30 did not contact the helpline as the first point of entry because they were unaware of, or could not remember that it was the national referral service in Ireland. A 34-year-old social worker explained: “My friend told me about MyOptions. Otherwise, I wouldn’t have known” (SU11). This participant rang MyOptions only after several steps: “No I never heard of MyOptions. I made an appointment with my GP who was not helpful; at all. …I saw the Rotunda [hospital] webpage said something about MyOptions at the bottom. I thought MyOptions was counselling only. I’m so glad I rang them.” Indeed, seven service users who were unaware of MyOptions called their own GPs first, learned their doctor did not provide, and were then referred to MyOptions.

Several service users indicated the need to boost advertising. For example, a 30-year-old homemaker in a rural area emphasised that she saw no advertisements and argued that, “[MyOptions] needs to be publicised more so that women know that they don’t have to wait a week for their GP if they are further along” (SU19). A 38-year-old family carer in rural Midlands also noted, “information should be at your GP’s though. If every GP did it [provided abortion information], it would be helpful. I was surprised that there are four GPs in the surgery and none of them are doing it” (SU26).

While the MyOptions helpline also provides critical information that the service is free of charge to Irish residents, the majority of women in our study were unaware that abortion care is free in Ireland; although some participants remembered the Repeal campaign slogan “Free, Safe, Legal.”

#### Strategy three: Modifications during the COVID-19 pandemic

Shortly after the onset of the COVID-19 pandemic in early April, 2020, the Irish government modified the model of care to permit remote consultations for both visits to reduce the time people would have to spend in GP surgeries, and facilitate access to care under pandemic travel restrictions. This modification did not require a change in legislation, but rather, in the interpretation such that the consultations can take place via remote service. The START organisation proposed telemedicine consultation protocols, which the HSE adopted when developing the modifications. A designated proxy was permitted to pick up the medication for the patient after the 3-day wait. The changes were publicised by the HSE national lead for abortion services via social media and Ireland’s main radio station [31]. The HSE if needed paid “for a taxi or a courier to take the medication from the GP and to deliver it to the lady” (KI8, KI9). Service users in this study who sought care during the pandemic had a favourable experience with the way telemedicine alleviated the burden of two in-person visits resulting from the mandatory 3-day wait, and appreciated access to a GP near them.

“I was able to access care via telemedicine. Like I said, I don’t have family here for support. And I have a daughter to take care of. She’s just one year old. Being able to do the consultations over the phone made it easier.”

(SU19)

Occasionally, a service user preferred visiting the clinic: “I didn’t access telemedicine. It felt more normal to go in person” (SU4), or did not mind doing so, “I was happy to go in” (SU27). The GPs generally valued seeing the patient for at least one in-person consultation and conveyed the importance of “a human-to-human conversation with them” (GP17) for the quality of care they provide. In-person visits also conveyed a sense of greater security for the GP’s clinical judgement:

“Yes, I have [modified the service during COVID-19], in keeping with the change and the access. So, visit one now is a telephone consult. Visit two, for my own certainty and in keeping with the wording in our legislation of examination, I do like to palpate for the fundus and visit two, we’re trying to minimise the amount of time the patient is with us, so I give as much information on the phone on visit one.”

(GP14)

The HSE key informants’ reported that MyOptions began to provide up to three names of GPs to callers (as compared to two names before the pandemic) to assist women with accessing GPs, and overall found that telemedicine facilitated access:

“because those consultations were happening over the phone or by video link, people were getting GPs appointments quite quickly. So I can certainly remember a number of women saying that they’ve got consultations the same day. So I think if anything, provision kind of increased or certainly people’s needs were met more immediately than they might have done otherwise.”

(KI13, KI14)

Overall, our study participants reported few delays in community EMA due to the COVID-19 pandemic, both because of the telemedicine option and the fact that most patients continued to present relatively early at 6–7 weeks of gestation even during the pandemic.

### III. Remaining challenges for GPs and service users

Our data identify four significant remaining challenges experienced by both GPs and service users in the way abortion services are being implemented in Ireland.

#### Unclear or slow referral pathways to hospitals

GPs are responsible for referring a patient who has reached 10 weeks gestation to secondary care, but the pathways to transfer patients are not always clear or reliable. The queries in early 2019 reported by the HSE reflected that GPs were asking “about exactly which [maternity] hospital is providing the service. ‘I’m ringing the number, there’s no one answering’” (KI3, KI4). As of 2021, the abortion services available in each maternity unit remain obscure and are mainly known to GPs through word of mouth. GPs reported that this lack of clarity on the formal pathways creates uncertainty that the pathway will be available or timely when they need to refer a patient. Some emblematic quotes from GPs working in or near large urban hospitals in the east and south of Ireland convey these concerns:

“…it is hard for patients and you know, this particular maternity hospital gives a very poor service and has a very poor attitude…. It’s a disaster. You have to send in the request by email. They won’t talk to you on the phone.”

(GP20)

The referral pathway to hospital is “not satisfactory, and it’s not satisfactory that…you know, if I had a medical problem…, I would be relying on my established relationship with those Consultants who I know provide from the campaigning work and from the designing of the guidelines and all of that stuff, …rather than knowing that there is a formal proper defined referral pathway. …if this midwife goes out sick for a period of time, or leaves, or finds another job, then there doesn’t seem to be a proper replacement. It works well as it is, but this is not good in the long term.”

(GP44)

In 2020, the HSE has taken steps to improve the pathway to hospitals. They “have emailed out to all the hospitals asking them for updated contact details for their Nurse Midwife Coordinator and the email addresses a referral needs to be sent to, just to make sure that it’s as consistent across the board as it possibly can be” (KI26).

#### Non-providing GPs: Unreliable referrals and obstructions

While service users in this study typically reported positive experiences with community abortion providers, they also described challenges with referral pathways. In particular, referral by their own non-providing GPs to abortion providers was unreliable, at times caused delays, or involved unpleasant encounters. For example: “My own GP was misleading. It didn’t need to be as upsetting as it was” (SU23), or “She tried to talk me out of getting an abortion. She asked me to get counselling which might help me [to start thinking about continuing with the pregnancy]” (SU15). This 43-year-old nurse, residing in a suburban area recounted:

“I went to my own GP unnecessarily. That delayed me by two days because I couldn’t get an appointment until then… She brought me in. She asked me the reasons why I was having a termination. Did I have a previous pregnancy?, all these questions. I felt like I was having to justify my decision. That was really hard. Then she went out of the room, came back and said we don’t provide the service here. I was stunned.”

(SU10)

Non-providing GPs who pose obstructions, including those who are objectors, may not always become barriers that prevent abortion access, but may nevertheless delay or protract patient journeys for service users seeking a lawful service.

#### Unreliable referral pathways for ultrasound scans

Our data also show that not all GPs have a reliable and timely pathway to access ultrasound scans. Scans are not routinely required and are therefore ordered at the discretion of the GP, but timely availability of this service is critical when the patient is near the legal limit for a community EMA. In 2018, the HSE contracted the private company Ultrasound Services to provide pregnancy dating scans. Since only six Ultrasound Services locations offer pregnancy dating scans in Ireland, some GPs (for example in the Midlands) must instead use local hospitals for the scans. Some Ultrasound Services locations lack adequate staffing with doctors complaining that “when that radiographer is off, there is no access to scans” (GP11), or fail to provide a timely service: “They’re very slow and very difficult to get hold of” (GP20). A feeling of uncertainly prevails for numerous providers as this rural doctor elucidates:

“…I don’t know how long it’s going to take for them to do them. So, if I referred a woman this afternoon, I don’t know whether she’ll have that scan tomorrow, or Saturday, or Sunday, or Monday, or when it will be. I’ve had a couple of problems in that I sent in scan requests say…on a Monday or Tuesday, and I get a phone call on the Thursday or Friday, we’ve got no Sonographer. Yes, and then I have to go back and try and source this somewhere else.”

(GP44)

In one case, a private radiology service refused to offer pregnancy dating scans altogether, despite their HSE contract:

“we have ultrasound services not too far away from us and that ultrasound services centre is not providing scans. So even though the contract has gone to Ultrasound Services through the whole of Ireland, individual sites have chosen whether they choose to undertake scans for terminations of pregnancy or not.”

(GP14)

Three of our service users were referred for scans when accessing EMA; two of them reported no challenges, and one had to be booked at a hospital after an Ultrasound Services clinic had no availability.

#### Unnecessary mandatory 3-day wait

The Irish model of abortion care requires a statutory 3-day wait after the first consultation, necessitating at least two visits. Both the GPs and service users in this study believed that this is unnecessary and can lead to delays. For example, this 30-year-old college student who sought an EMA in the west of Ireland explained, “The only delay is the three day wait. For me it was not an issue because I was so early, but for those who are on the cusp it must be very stressful” (SU7). While all but a single GP wanted this legal requirement lifted or to make it optional, several GPs also mentioned that there can be usefulness to the wait for some patients, for example:

“So, it would be nice to have a little flexibility about that, you know, because usually when women have showed up, they’re pretty 100% sure like that they want to go ahead. … it would be nice to shorten it to 24 hours or have the option, you know.”

(GP17)

“So I think the little delay, the three-day delay, for me, it’s actually a good thing. The only part that’s really bad is when she’s coming up to 12 weeks. Then it’s a disaster because you can be in an awful situation, especially if the hospital are being difficult, you know.”

(GP20)

Some participants questioned the rationale for the 3-day wait, given that most women have already made their decision:

“I think it’s quite paternalistic that we have decided that this three-day waiting here needs to be there, and I just don’t really understand it because I don’t think we take that attitude to much other healthcare.”

(GP44)

“Yes. I think that’s infantilising women. I think when they see a GP, the majority have decided and GPs are extremely good at spotting people who are undecided.”

(KI20)

The mandatory 3-day wait and the strict 12-week gestational limit also contribute to providers’ fears of potential legal repercussions when the patient is nearing the limit. GPs expressed concerns about patients who commenced the process but should they have a failed EMA may not be able to complete their care before the 12 week limit. For example, “The big thing is for us, if we’re providing close to the 9 + 6 days is that we follow them up very closely to make sure that it’s completed… You don’t want them to go over 12 weeks” (GP12). Another GP explained, “I think there’s also a worry that, will women get complications?… There’s that fear and the litigation aspect of it as well” (GP7). From a legal perspective, “it’s very criminalised thinking and it’s very rigid” which diverts attention from focusing “on harms to women and positive health outcomes for women” (KI1).

#### Uneven access for migrants

One of the more common queries from GPs directed to the HSE have been about the ineligibility for state-subsidised abortion care of migrants who lack a Personal Public Services number (PPSN), which entitles Irish residents to social welfare, including public health services. Interviews with service users clearly illuminate this problem. Migrants lacking the PPSN or those waiting for the government to issue one have to pay out-of-pocket for at least part of the treatment. “I didn’t have a PPS number so the doctor informed me that I’ll have to pay a deposit of €100 that can be refunded once I get the PPS number… The PPS number would have been an obstacle if I didn’t have the €100 deposit” (SU9). We found that GPs and MyOptions try to accommodate these patients with individualised management of cases. The experience of a college student in Ireland who is a UK national exemplifies this problem:

“With the PPS issue she [MyOptions] said if Dr. X won’t do it then get back to me and we will find someone who will. It could have been much worse if people hadn’t been so nice to me. I had applied for a PPS number and I had an email receipt of that. Dr. X spoke to the HSE and was told he has 3 months to make his claim with them. It takes 8 weeks for the PPS number. Maybe they could work something out with the European Health Insurance Card? If I hadn’t had a doctor that was so nice this could have been a huge obstacle. The receptionist in Dr. X’s office said you need to come back in eight weeks when you have your PPS number. I was worried about this. Then I got a call from Dr. X who said I don’t need to come back and not to worry about it.”

(SU7)

The lack of PPSN also makes it difficult for providers to receive payment for services, because the HSE reimbursement scheme is based on the patient’s PPSN.

Migrants who need translation assistance and their GPs may also encounter challenges. While MyOptions has interpreters available for initial information, the HSE translation service is not always available when patients are in the doctor’s office. Some GPs use Google translate to communicate with patients when needed. For example, one GP explained, “this is an issue where the HSE do fall down. They are supposed to provide interpreters for us but that doesn’t always happen, and it’s not always possible to get an interpreter when you need one” (GP1). Another GP who sees patients from Latin America observed that “that [translating] is one of the things that is more difficult over the phone…. The HSE has a service of translators, but it’s not accessible for us” (GP28). In general, GPs improvise using online translation websites. Some patients bring a relative, a friend or a co-worker to assist in translation, but clinical guidance for providers advise that this is not best practice [30].

#### Uneven distribution and shortage of services

At the time of our study there were 385 GP providers based in general practice in 25 of 26 counties, and in five women’s health clinics in Dublin, with County Sligo lacking a provider altogether. However, an uneven distribution and incomplete geographic coverage of abortion services both in communities and in hospitals remains in some areas, in particular rural regions and in the west and north of Ireland. These gaps in geographic coverage were acknowledged by the HSE which reported that that “there were various reasons, but one of them was because they didn’t have the hospital backup” (KI26). A informant from the medical community confirmed: “If you happen to be a GP that’s essentially two hours away from your nearest referral centre, that’s a huge deterrent to providing the service… if there’s a failure, you don’t have a centre that’s definitely guaranteed to take that person and that’s what everybody dreads… that’s a very alone place to end up as a GP” (KI6). Geographic gaps were also reflected in the experiences of GPs who reported from 12 of the 26 counties, as well as from service users in our study.

For example, a 39-year-old teacher in the Midlands who was over 9 weeks gestation reported that “There aren’t any providers in my town which has a population of 30–40,000 people” and “I mean I did have to get buses and all to go in and out of the GP and the hospital” (SU27). Others residing in rural and suburban areas noted,

“I live in a rural community. There are no providers within commuting distance. The closest is 30 minutes drive away. There are not enough providers in the area.”

(SU23)

Inadequate public transport can pose serious problems:

“My partner drove me to the GP practice. I am very lucky really. The drive from my home to the GP is 30 minutes. If I didn’t have a car or had no one to drive me that would be the biggest obstacle.”

(SU7)

This interviewee suggested that “maybe a partially funded taxi service” can be offered for women who have to travel.

Echoing women’s experiences, the GPs reported seeing patients who travelled from a wide range of counties. Asylum seekers housed in direct provision centres around Ireland face particular challenges with transportation:

“I have seen a woman who was living in direct provision who had to get a bus to Cork and a bus back to [town] to see me, and she had a very clear and definite decision and I saw her long before the COVID thing, so the remote provision wasn’t an option for her, and I felt very sorry for her…. It was a huge imposition on her.”

(GP44)

A GP located in a more sparsely covered region reported that 80–90 per cent of patients seeking abortion at his rural clinic are travelling from the more populated parts of the county that lack a provider. Overall, he believed the problem is not that there are insufficient GPs providing care in Ireland, but rather a highly uneven distribution, leaving rural sections and areas with poor public transport underserved.

Being overworked presented a constant challenge for GPs in our study, which may discourage them from taking up a new service, a problem that is especially acute for rural clinics. These excerpts are emblematic of this problem:

“[The] main challenge is the nature of general practice. It’s just gotten very, very extremely busy, and we have had our funding cut during the recession, which is only starting to be restored. So, people have felt very snowed under and may have no issue at all about it, but it does take up, they’re long consultations, they do take up time and you, they feel that they’ve no more space in their life to take on an extra role. So yes, I think that’s the biggest barrier.”

(GP11)

Task sharing of abortion provision with midwives or nurses who are already part of community clinics’ staff has arisen in some interviews as a potential solution, as this GP explained,

“It would lessen my load, because a lot of the first visits is literally explaining to the woman what’s going to happen and what’s involved and what the legal requirement and all that is, and really I am not needed for that in many ways.”

(GP44)

One provider argued that, “in keeping with that very important concept of task shifting, I believe that practice nurses actually would probably be better than GPs” (GP6).

On the other hand, many also expressed reservations about potentially declining patient flow if the number of providers in the country grows, especially in the areas with good GP coverage. Their main fear was the need to maintain skills, “I’m not sure that 2,000 doctors in the country, all doing one termination a month is necessarily good care. You know, you need to be doing it regularly to be doing it right” (GP1). Some expressed concerns about getting “rusty” with only infrequent patients:

“…at the moment at about one per week, actually, I find that an appropriate number insofar as like any skillset, if you’re not doing it on a regular basis, you become rusty and you forget what questions you need to be asking, all those sort of things. So, the amount of terminations we’re doing are probably appropriate in order to keep the skillset up, but not overwhelm us capacity-wise.”

(GP15)

Overall, the availability of abortion care across Ireland is a debate that encompasses not only the issue of the availability and geographical dispersal of providers, as well as questions of what is an “appropriate” number of patients for GPs to maintain confidence in their skills, but remain within their work capacity.

## Discussion

This article analyses implementation of the abortion policy in Ireland with a focus on the community model of care. We found that the community model is a successfully implemented service, within the parameters of the current law and guidelines.

Accessibility of the services stands out as an overarching and explicit goal for both the Irish government and general practitioner medical community. This is evident in the choice to embed medical abortion care in community-based GP practices as the backbone of the Irish abortion provision. The decision to make abortion services accessible in the wide network of community clinics via medical abortion proved to be a productive approach given its suitability for a rapid roll-out. Studies with Irish GPs in the years before the policy change showed that most doctors supported abortion provision in certain situations [32], and supported their patients’ decisions to seek abortion [33]. Moreover, medical abortion entails less training than surgical abortion and can be incorporated with relative ease into the existing primary care infrastructure [34]. This infrastructure in Ireland is substantial: there are approximately 3500 GPs across 26 counties, and an average Irish person visits their GP more than four times per year [35].

Our study also revealed a strong political will at the highest level of health policy-making, namely the Minister for Health, and Department of Health, and the HSE, to implement the abortion policy. Political will has been identified by scholars in other geopolitical contexts as a decisive ingredient in making abortion access a reality [34]. The political time pressure proved to be a significant facilitator and by 1 January, 2019, the service in many community clinics was ready and commenced across Ireland, even if only nine of 19 hospitals commenced abortion provision at that time [36]. Since then, the rising number of GP providers, the embeddedness of abortion in primary care, and the sheer fact the services have been running without interruptions (including during the pandemic) reflects an increasing normalisation of this healthcare service, even as challenges to complete accessibility remain. The leadership role of dedicated GPs also demonstrates political will as engaged and often visible doctors, midwives, and nurses went out on a professional limb to show support for abortion rights, create solidarity, and work toward normalisation of the service.

The modifications to allow telemedicine during the COVID-19 pandemic maintained accessibility of abortion care by reducing problems with transportation, and were received positively by service users and providers alike. Therefore telemedicine services should be continued beyond the pandemic to reduce in-person visits and the logistical and economic burdens for patients, thereby facilitating access to care. Of note, some GPs nevertheless prefer to see the patient for the second consultation, as they see human contact and interactions contributing to patient-centered quality of care. However, the fears expressed by providers regarding missing a clinical sign and potential legal repercussions when the patient is nearing the 12-weeks gestational limit may also play a role in this preference, and may in part be fueled by the fact that abortion outside of the current legislation continues to be criminalised in Ireland. Additionally, providers’ concerns about maintaining skills in abortion provision (or fears of de-skilling when not providing procedures on regular basis) may also reflect the chilling effect of criminalisation. Providers’ concerns about skills may be addressed, in part, through regular opportunities for refresher and new training provided via the ICGP, START, and other professional organisations. The international human rights bodies also recognise criminalisation’s chilling effect on abortion care provision and the need to decriminalise abortion legislations [37].

While abortion helplines are used in variety of ways around the world [38], the Irish MyOptions helpline stands out as the key structural facilitator making abortion accessible through a single, centralised portal of entry for accessing care anywhere in Ireland (except Sligo, which lack providers). The safeguarding of the provider list by the HSE and MyOptions highlights the government’s commitment to provider confidentiality. The recognition of the need for privacy and confidentiality is an important form of support for providers that is built into the Irish model of care. It shields providers in a sociocultural and political context in which abortion stigma continues to play a substantial role [39, 40].

Our study clearly showed that once women contacted the helpline, their experience was generally positive and led to a swift referral to a viable provider. This service circumvents potential problems of non-referral from a non-providing or “objecting” GP. However, a remaining challenge is the relatively low awareness among service users of MyOptions as the portal of entry for abortion care. To address this gap, the HSE should continue to dedicate funding to expand and sustain MyOptions advertising, and the ICGP should consider encouraging all GPs to list MyOptions’ contact on their clinics’ websites. Underutilisation of MyOptions has led to disturbing experiences with non-providing GPs, some of whom object to abortion. This is especially urgent as service users who are unaware of MyOptions are likely to contact their own GPs first. Our study highlights the need for the Irish Medical Council to clarify the ethical obligation of objectors and non-providers to offer accurate information and timely referrals for women who seek abortion services, to disseminate this information by working with providers, and to regularly monitor whether these ethical obligations are met. These obligations are already stated in the Guide to Professional Conduct and Ethics [41], which was specifically amended to address abortion policy implementation. Other information gaps include concerns about access to care for migrants who need the PPSN to receive the service free of charge but may not be aware of this requirement, as well as lack of reliable translation services during medical visits. Lastly, while MyOptions is an efficient switchboard managing the flow of patients to GPs, the fact that women who are past the 9 weeks plus 6 days gestation must still be funneled to GPs rather than directed to hospitals where they will receive care (or be able to self-refer there) raises concerns about contributing to delays. Also, timely and appropriate ultrasound scans should be ensured when needed. We further recommend that the HSE standardise and ensure reimbursement of GPs for care to patients without PPSN, and establish a reliable interpreter service for providers and patients, including translation of medication labels.

The Irish community model for EMA follows the WHO guidelines [42] to facilitate more optimal use of resources, by empowering individuals who choose to self-manage their abortion process. However, barriers remain, including the statutory requirement for a 3-day wait [43], which automatically delays care and should therefore be eliminated.

Finally, our study clearly captured the problem of GPs’ excessive workloads, a deterrent for new doctors, especially in rural areas, to take on provision of abortion. This issue speaks to the need to expand the number of providers outside of the main urban centres. A recent study measuring GPs’ real-time workload [44] showed that GPs work extra-long hours, and one-third of their work time is non-remunerative, and their pay had been cut by approximately 40% as part of austerity measures during the 2008 financial crisis and is yet to recover. These factors present challenges for recruiting and retaining GPs into practices. An ICGP report with a plan for improving primary care highlighted problems for rural areas, including shortages of doctors interested in rural practice and difficulties in recruiting locums [45]. Sustainability of this workload, especially in rural practices, is challenging, therefore adding an abortion service, in particular if the GP goes on the public MyOptions list (which generates a higher volume of patients) may present an unsurmountable added burden. Dedicating more state resources to support general practice is vital for the long-term sustainability of abortion care.

This problem is aggravated by the legislation which requires a GP to conduct both consultations with service users, and does not allow other health professionals to provide abortion care. The WHO guidelines on health worker roles and task-sharing assert that “[p]lanned and regulated task shifting and task sharing can ensure a rational optimisation of the available health workforce, address health system shortages of specialised health-care professionals” [46]. Task sharing with midwives and/or nurses who normally work in community surgeries alongside GPs may be an important next step in easing the workload burden currently experienced by Irish GPs. Ultrasound scanning shortages which our study identified could also be addressed by expanding qualification to date pregnancies to midwives and nurses through training in first trimester scans [47]. This shift would require a re-evaluation by the HSE of their training scheme and credentialing requirements.

The unreliable or unclear referral pathway to secondary care also discourages some GPs from becoming abortion providers. This problem may be especially pronounced in rural regions with few hospitals. Without clear backup from a local hospital, provision of EMA may seem too risky to some doctors. Sligo in particular remains without a provider, despite the county’s clear vote in favour of the Repeal in 2018 [48]. While pathways between primary and secondary care are typically established by hospitals, the HSE and its national lead for abortion services may have a role to facilitate and standardise the development of these protocols.

## Limitations and strengths

It is important to note the study’s strengths and limitations. This study’s overarching objective is to be informative about barriers and facilitators of the abortion policy implementation in Ireland, therefore these findings may not apply to other countries. The strength of the study is the triangulation of data from three samples, and the wide geographic distribution of participants in the provider and service user samples. Our team was mindful of the potential for selection bias with purposive sampling, therefore we sought to minimise this limitation by inviting participation from as many geographic and stakeholder communities as possible. Still, we experienced challenges in recruiting some marginalised populations of service users. Specifically, despite multiple attempts to collect data with women in the Irish Traveller community and those living in Direct Provision we were unable to reach these populations. Further research is needed to examine the perspectives of these groups.

## Conclusions

Overall, this study shows that the Irish community model of care via medical abortion provision delivered by GPs has been successfully implemented through collaborations between the government and the medical community, GPs’ leadership, and supports for providers and service users. The community delivery of care and the national helpline constitute key features that could be duplicated in other contexts and countries. The remaining challenges can be addressed with continued political will to improve service implementation, in particular through the elimination of potential delays, and the expansion of credentialing of healthcare professionals to share in provision of this service. Legislative barriers to accessing legal abortion care in communities such as the 3-day wait should be removed to allow for full implementation of legal community-based abortion services.

## Supporting information

S1 Appendix SRQR checklist. (PDF)
